# Pregabalin: Potential for Addiction and a Possible Glutamatergic Mechanism

**DOI:** 10.1038/s41598-019-51556-4

**Published:** 2019-10-22

**Authors:** Yusuf S. Althobaiti, Atiah Almalki, Hashem Alsaab, Walaa Alsanie, Ahmed Gaber, Qasim Alhadidi, Ana Maria Gregio Hardy, Abdulrahman Nasr, Omar Alzahrani, Creed M. Stary, Zahoor A. Shah

**Affiliations:** 1 Taif University, College of Pharmacy, Department of Pharmacology and Toxicology, Taif, Saudi Arabia; 2 Taif University, College of Pharmacy, Addiction and Neuroscience Research Unit, Taif, Saudi Arabia; 3 Taif University, College of Pharmacy, Department of Pharmaceutical chemistry, Taif, Saudi Arabia; 4 Taif University, College of Pharmacy, Department of Pharmaceutics and Pharmaceutical Technology, Taif, Saudi Arabia; 5 Taif University, Faculty of Applied Medical Sciences, Department of Clinical Laboratories Sciences, Taif, Saudi Arabia; 6 Taif University, Faculty of Sciences, Department of Biology, Taif, Saudi Arabia; 7 Department of Anesthesiology, Perioperative and Pain Medicine, Stanford Medical School, Stanford University, CA, USA; 8 Department of Physiology and Pharmacology, College of Medicine and Life Sciences, University of Toledo, OH, USA; 9 Department of Medicinal and Biological Chemistry, College of Pharmacy and Pharmaceutical Sciences, University of Toledo, OH, USA

**Keywords:** Medical research, Neuroscience

## Abstract

Drug addiction remains a prevalent and fatal disease worldwide that carries significant social and economic impacts. Recent reports suggest illicit pregabalin (Lyrica) use may be increasing among youth, however the addictive potential of pregabalin has not been well established. Drug seeking behavior and chronic drug use are associated with deficits in glutamate clearance and activation of postsynaptic glutamatergic receptors. In the current study, we investigated the abuse potential of pregabalin using conditioned place preference (CPP) paradigm. Different doses of pregabalin (30, 60, 90, and 120 mg/kg) were used to assess the seeking behavior in mice. Glutamate homeostasis is maintained by glutamate transporter type-1 (GLT-1), which plays a vital role in clearing the released glutamate from synapses and drug seeking behavior. Therefore, we investigated the role of glutamate in pregabalin-seeking behavior with ceftriaxone (CEF), a potent GLT-1 upregulator. Mice treated with pregabalin 60 and 90 mg/kg doses demonstrated drug seeking-like behavior, which was significantly blocked by CEF pretreatment. These results suggest that pregabalin-induced CPP was successfully modulated by CEF which could serve as a lead compound for developing treatment for pregabalin abuse.

## Introduction

Addiction to several drugs and substances remains a critical health issue worldwide. According to the world drug report, Saudi Arabia had one of the highest reported drug seizure rates in 2011^1^. As stated in several reports, the prevalence of drug addiction is high in Saudi Arabia, as are associated rates of hepatitis, HIV, crime, and socioeconomic decline (for review see^2^).

Illicit Pregabalin (Lyrica) use has recently become more common among young Saudis and worldwide^3–10^, likely secondary to lack of appropriate regulation. Pregabalin is an analogue of the neurotransmitter gamma-aminobutyric acid (GABA) that can bind with strong affinity to the α2δ subunit of pre-synaptic voltage-gated calcium channel receptor to decrease post-synaptic excitatory neurotransmitter release^11^. Pregabalin has a therapeutic value in the management of fibromyalgia, generalized anxiety disorder, diabetic neuropathy, partial epilepsy, and postherpetic neuralgia^12–14^. There remains controversy regarding the abuse potential of pregabalin in preclinical studies. In conditioned place preference (CPP) studies conducted in rats, pregabalin (up to 30 mg/kg) did not cause rewarding effects and did not change place preference^15,16^. However, these doses were small compared to higher doses used in previous case reports of pregabalin abuse^4–9^. Interestingly, pregabalin has been reported to cause euphoric effects as a side effect in participants of controlled clinical studies^17–20^.

Importantly, glutamate is the major excitatory neurotransmitter in the brain. The nucleus accumbens (NAc) is involved in the reinforcing and rewarding effects of several drugs of abuse^21–23^. Disturbances in glutamate homeostasis in the NAc have been shown to be associated with drug seeking behavior and chronic drug use^24–28^. The release of glutamate from prefrontal cortex (PFC) projections into the NAc has been shown to mediate drug seeking behavior in previous studies^29,30^. Blocking glutamatergic activation of mGlu1^31^, mGlu5^32,33^, AMPA receptors along with blocking the release of glutamate in the NAc was found to reduce reinstatement of self-administration for different drugs of abuse^30,34–36^. It is noteworthy that glutamate homeostasis is controlled by a number of glutamate receptors and transporters. One of these transporters is glutamate transporter type-1 (GLT-1) which can clear the majority of the synaptic glutamate^37–40^. Moreover, treatment with ceftriaxone (CEF), a well-known upregulator of GLT-1 expression, has been shown to oppose drug-seeking behavior induced, in part, by downregulation of GLT-1 following exposure to several drugs of abuse^26,41–45^. Therefore, in the present study we explored the abuse potential of different doses of pregabalin using CPP and assessed the potential mechanistic role of GLT-1 in pregabalin associated drug-seeking behavior by pretreatment with CEF.

## Materials and Methods

### Animals

Male BALB/c mice, weighing 25–30 g were obtained from King Fahd Medical Research Center (KFMRC, Jeddah, SA). Mice were housed in standard cages with controlled constant temperature (21 °C) and humidity (30%) on a 12:12 light-dark cycle. Animals had *ad libitum* intake of water and food. Mice were allowed to habituate for seven days before conducting any experiments. The experimental procedures of animals were ethically approved by the Research Ethics Committee at Taif University, in accordance with the Institutional Animal Care and Use Committee of the National Institutes of Health guide.

### Drugs

Pregabalin was generously provided by Jamjoom Pharmaceuticals (Jeddah, KSA). Ceftriaxone was gifted by King Abdulaziz Hospital at Taif. All the drugs used in this study were reconstituted in sterile saline solution (0.9% NaCl).

#### Experimental design

**Figure 1:**
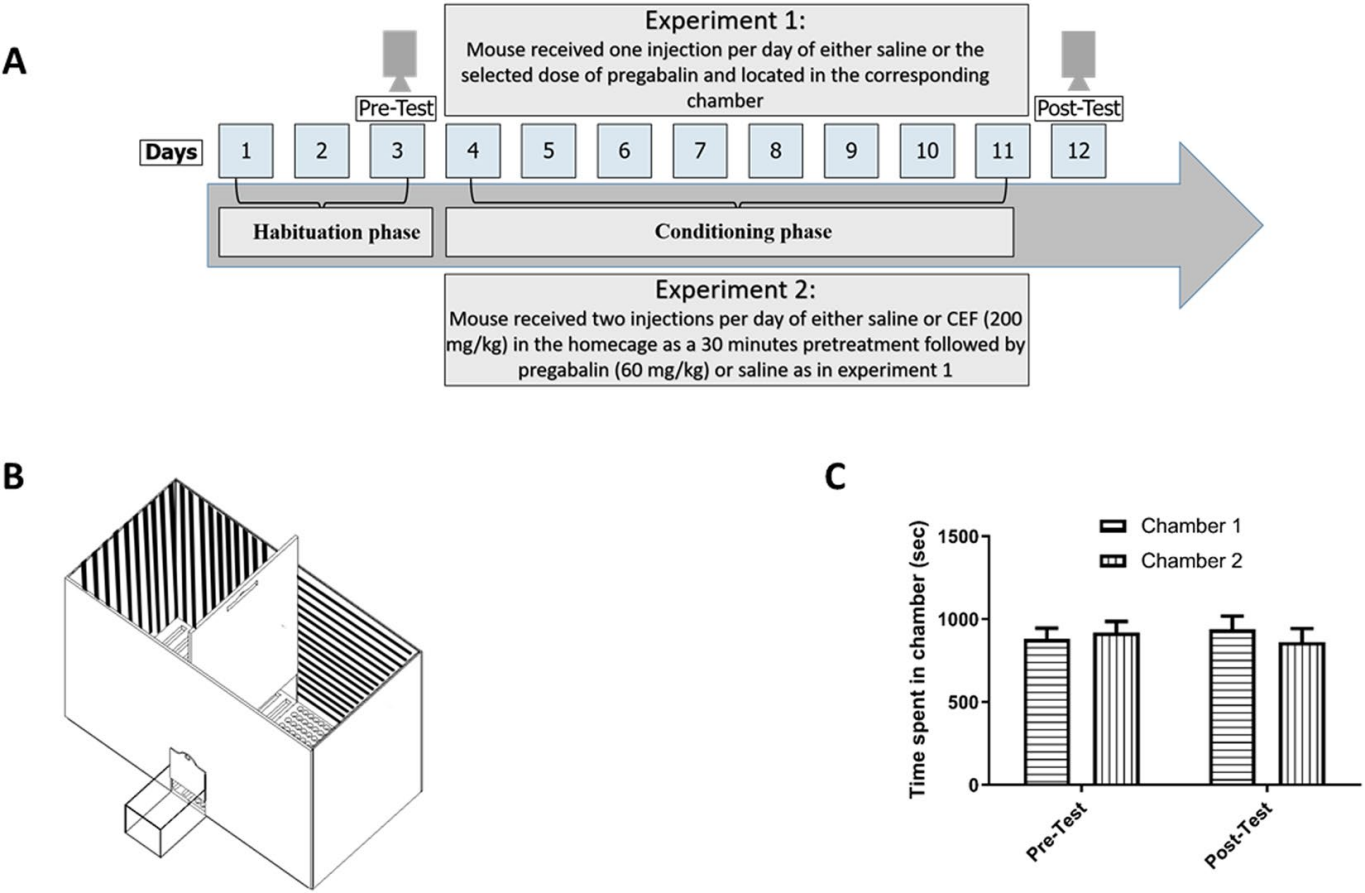
Experimental design of the CPP experiment 1 and 2 (**A**). Diagram of the CPP apparatus (**B**). Time spent in chamber 1 and 2 in the control group (**C**). No significant changes in time spent were found in chamber 1 and chamber 2 during the tested phases. Values shown as means ± S.E.M.

The overall experimental design is presented in Fig. 1A.

Experiment 1: Mice were randomly assigned into five groups; Group 1: Control group (n = 8), mice in this group were treated with vehicle for 8 days. Group 2: Preg-30 group (n = 8), mice were injected with pregabalin (30 mg/kg, i.p.x 4) and vehicle for eight days during the acquisition phase. Group 3: Preg-60 group (n = 8), mice were injected with pregabalin (60 mg/kg, i.p.x 4) and vehicle for eight days during the acquisition phase. Group 4: Preg-90 group (n = 8), mice were injected with pregabalin (90 mg/kg, i.p.x 4) and vehicle for eight days during the acquisition phase. Group 5: Preg-120 group (n = 8), mice were injected with pregabalin (120 mg/kg, i.p.x 4) and vehicle for eight days during the acquisition phase. Mice were then examined for place preference following completing the conditioning training.

Experiment 2: Mice were randomly assigned into four groups; Group 1: C-C group (n = 8) Mice in this group were treated with vehicle in home cage as a 30 minutes pretreatment and vehicle for eight days during the acquisition phase. Group 2: CEF-C group (n = 8) Mice were injected with CEF (200 mg/kg, i.p.) in home cage as a 30 minutes pretreatment and then vehicle for eight days during the acquisition phase. Group 3: C-Preg group (n = 8) Mice were treated with vehicle in home cage as a 30 minutes pretreatment and then Pregabalin (60 mg/kg, i.p.x4) as well as vehicle for eight days during the acquisition phase. The dose of Pregabalin which induced CPP in the previous experiment was used in this study. Group 4: CEF-Preg group (n = 8) Mice were injected with CEF (200 mg/kg, i.p.) in home cage as a 30 minutes pretreatment and then Pregabalin (60 mg/kg, i.p.x4) as well as vehicle for eight days during the acquisition phase. Mice were then examined for place preference following completing the conditioning training.

#### Conditioned place preference paradigm

A custom made acrylic CPP apparatus (Fig. 1B) was used in this study as described in our previous work^46^. Briefly, this apparatus consists of two equal-sized conditioning chambers (35 cm × 35 cm × 50 cm), and one start box (10 cm × 15 cm × 10 cm) located outside of the CPP apparatus. The two conditioning chambers are distinguished by both tactile and visual cues. The interior walls of the first chamber are white in color with horizontal black stripes and textured walls (chamber 1). The interior walls of the other chamber (chamber 2) are black in color with vertical white stripes and smooth walls. The floor of chamber 1 is perforated with round holes. The floor of the other chamber is perforated with rectangle holes.

Habituation phase: The preconditioning day is considered as day one. On days one, two, and three each mouse was placed in the start box with door closed for 3 minutes. Then, the door was opened to let the mouse explore the conditioning chambers for 30 minutes. On day three, the animal exploration in both conditioning chambers was recorded by a digital camera fixed on the top of the apparatus. The time spent in both chambers was calculated using ANY-maze software (Stoelting, USA).

Conditioning phase: An un-biased CPP design has been used. Therefore, in each treatment group, half of the animals were randomly assigned to receive pregabalin and were placed in chamber 1, while the other half received this drug and were placed in chamber 2 during the conditioning phase (days four to eleven). Each mouse received intraperitoneal injection of specific treatment and were then located in the corresponding chamber with the door closed for 30 minutes session. On the following day, each mouse received vehicle and was located in the other chamber with the door closed for 30 minutes. The process was repeated until the completion of the eight conditioning sessions.

On day twelve, each mouse had free access to both chambers for 30 minutes. The time spent by the mice in both chambers was documented using digital camera (post-conditioning test) and counted by ANY-maze video tracking system.

### Statistical analysis

Two-way repeated measure ANOVA, (Phase × Treatment) was used to analyze time spent, at two different timepoints (pre-test and post-test), in the conditioning chambers in response to the selected dose of pregabalin or saline. This analysis was selected based on previous published work^46–49^. When significant main interactions or effects were found, Newman-Keuls multiple comparisons were performed. All data were statistically analyzed by GraphPad Prism, using a 0.05 level of significance.

## Results

### Experiment 1

**Figure 2:**
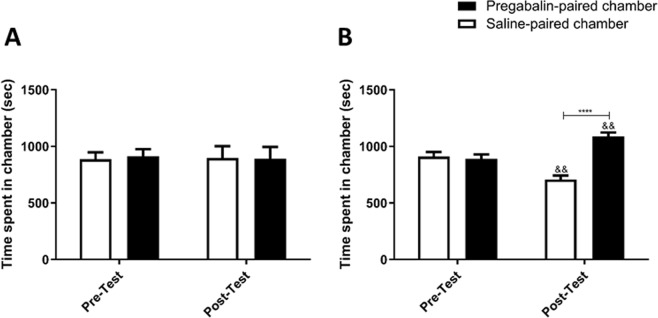
Time spent in pregabalin-paired chamber as compared to saline-paired chamber in Preg-30 group (**A**). No significant changes in time spent were found in the tested chambers. Time spent in pregabalin-paired chamber as compared to saline-paired chamber in Preg-60 group (**B**). A significant increase in time spent was found in pregabalin-paired chamber as compared to saline-paired chamber during post-test. A significant increase in time spent was found in pregabalin-paired chamber during post-test as compared to pre-test. Values shown as means ± S.E.M. ****p < 0.0001 compared to saline-paired chamber. (^&&^p < 0.01 compared to pre-test).

In this experiment, the abuse potential of different doses of pregabalin was assessed. No significant effect was revealed when mice were treated with saline in the conditioning training in phase [F (1, 7) = 0.1697, p = 0.6927], treatment [F (1,7) = 0.08970, p = 0.8965], and interaction either over phase and between treatments [F (1, 7) = 0.0006396, p = 0.9805; Fig. 1B]. After treatment with 30 mg/kg pregabalin throughout the conditioning phase, no significant effect was observed over phase [F (1, 7) = 5.282, p = 0.0551], or between treatments [F (1,7) = 0.02283, p = 0.8842], and interaction between phase and treatment [F (1, 7) = 0.01197, p = 0.9160; Fig. 2A].

No significant effect was observed between mice when treated with 60 mg/kg pregabalin throughout the conditioning training over phase [F (1, 9) = 2.215, p = 0.1709], however, we observed a significant effect between treatments [F (1,9) = 6.929, p = 0.0273], and an association between phase and treatments [F (1, 9) = 38.02, p = 0.0002]. Post-hoc analysis demonstrated that time spent was significantly elevated in in pregabalin-paired chamber in comparison with saline-paired chamber in the post-test (p < 0.0001; Fig. 2B), and time spent was significantly increased in pregabalin-paired chamber in the post-test compared to the pre-test (p < 0.01; Fig. 2B).

**Figure 3:**
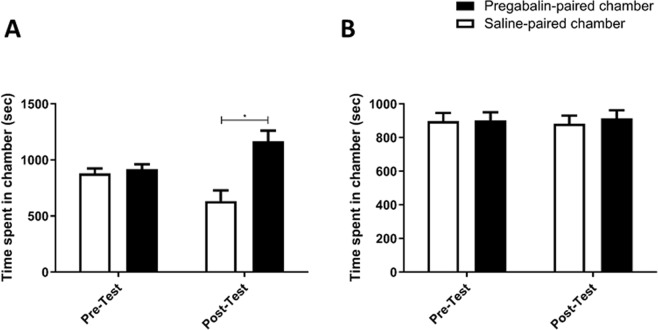
Time spent in pregabalin-paired chamber as compared to saline-paired chamber in Preg-90 group (**A**). A significant increase in time spent was found in pregabalin-paired chamber as compared to saline-paired chamber during post-test. Time spent in pregabalin-paired chamber as compared to saline-paired chamber in Preg-120 group (**B**). No significant changes in time spent were found in the tested chambers. Values shown as means ± S.E.M. *p < 0.05 compared to saline-paired chamber.

No significant effect was observed between mice when treated with 90 mg/kg of pregabalin during the conditioning training over phase [F (1, 8) = 1, p = 0.3466], but a significant effect was observed between treatments [F (1,8) = 7.195, p = 0.0278], and a significant association was observed over phase and between treatments [F (1, 8) = 6.036, p = 0.0395]. Post-hoc analysis revealed time spent in the pregabalin-paired chambers was significantly elevated in comparison to saline-paired chamber in the post-test (p < 0.05; Fig. 3A).

Administration of 120 mg/kg of pregabalin through the conditioning phase showed no significant change over phase [F (1, 10) = 0.8837, p = 0.3693] or between treatments [F (1,10) = 0.04083, p = 0.8439], and there was no considerable association between phase and treatment [F (1, 10) = 0.09504, p = 0.7642; Fig. 3B].

### Experiment 2

**Figure 4:**
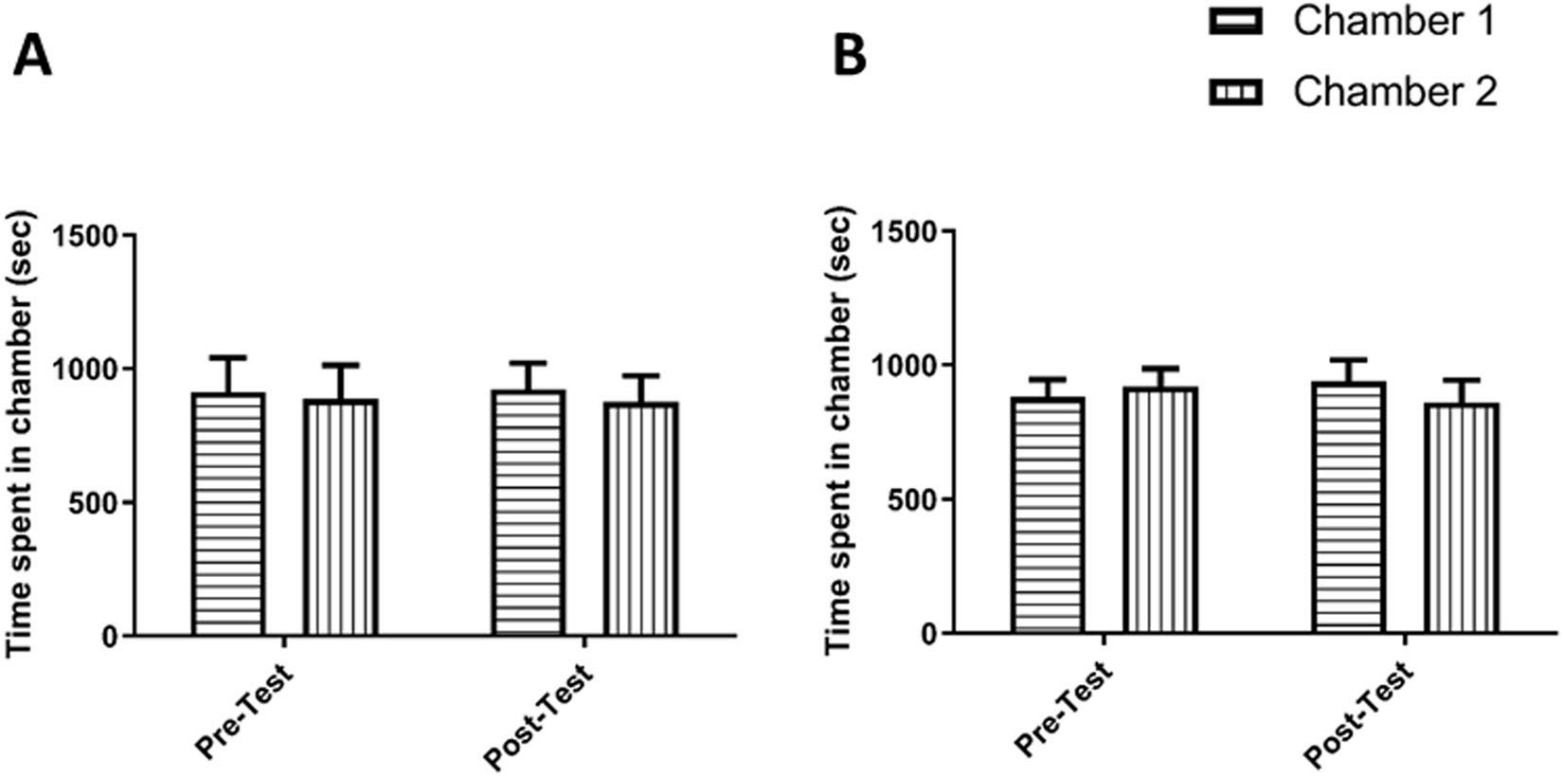
Time spent in chamber 1 and 2 in the C-C group (**A**). No significant changes in time spent were found in chamber 1 and chamber 2 during the tested phases. Time spent in chamber 1 and 2 in CEF-C group (**B**). No significant changes in time spent were found in the tested chambers. Values shown as means ± S.E.M.

In this experiment, the effects of CEF pretreatment on pregabalin (60 mg/kg) was assessed. When mice were treated with saline in home cage followed by saline treatment during the conditioning phase, no significant effect over phase [F (1, 5) = 5, p = 0.0756], between treatments [F (1,5) = 0.02791, p = 0.8739], or interaction between phase and treatment [F (1, 5) = 0.03735, p = 0.8544; Fig. 4A] were observed.

When mice were treated with saline after 30 minutes of treatment with 200 mg/kg CEF, in home cages, no significant effect over phase [F (1, 7) = 1, p = 0.3506], between treatments [F (1,7) = 0.0.01810, p = 0.8968], or between phase and treatment [F (1, 7) = 0.7249, p = 0.4227; Fig. 4B] was observed throughout the conditioning phase.

**Figure 5:**
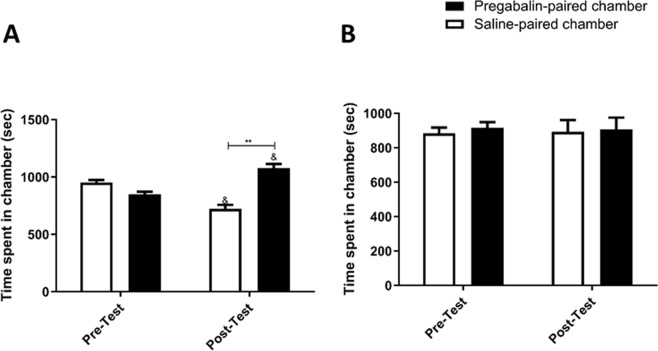
Time spent in pregabalin-paired chamber as compared to saline-paired chamber in C-Preg group (**A**). A significant increase in time spent was found in pregabalin-paired chamber as compared to saline-paired chamber during post-test. A significant increase in time spent was found in pregabalin-paired chamber during post-test as compared to pre-test. Time spent in pregabalin-paired chamber as compared to saline-paired chamber in CEF-Preg group (**B**). No significant changes in time spent were found in the tested chambers. Values shown as means ± S.E.M. **p < 0.01 compared to saline-paired chamber. (^&^p < 0.05 compared to pre-test).

Mice treated with saline in home cage followed by 60 mg/kg of pregabalin treatment during the conditioning phase; we did not observe any significant effect over phase [F (1, 6) = 1, p = 0.3559], however we did observe a significant effect between treatments [F (1, 6) = 7.605, p = 0.0330], and a significant interaction between phase and treatment [F (1, 6) = 39.63, p = 0.0007]. Post-hoc analysis demonstrated a significant increase in time spent in the pregabalin-paired chamber as compared to the saline-paired chamber in the post-test (p < 0.01; Fig. 5A, and time spent in pregabalin-paired chamber was significantly increased in the post-test compared to the pre-test (p < 0.05; Fig. 5A).

Treatment with 60 mg/kg of pregabalin after 30 minutes of treatment with 200 mg/kg of CEF, in home cages, resulted in no significant effect over phase [F (1, 8) = 1, p = 0.3466], between treatments [F (1,8) = 0.07929, p = 0.7854], or between phase and treatment [F (1, 8) = 0.01454, p = 0.9070; Fig. 5B] throughout the conditioning phase.

## Discussion

In the present study, we demonstrate for the first time, using a mice model of drug addiction, that pregabalin can induce CPP. These findings are in contrast to previous reports, however, the maximum tested dose of pregabalin was 30 mg/kg, which did not induce rewarding effects and did not change place preference^15,16^. Consistent with the previous findings from Andrews, *et al*., the dose of 30 mg/kg did not change place preference in our study. However, when the dose was increased to 60 mg/kg, a significant place preference was induced by pregabalin. This suggests a dose dependent effect of pregabalin’s rewarding effects. Interestingly, this effect is supported by previous controlled clinical studies^17–20^ showing that pregabalin can cause euphoric effects as a side effect in participants of these studies.

The drug seeking effects found in several drugs of abuse have been consistently reported to be mediated by glutamatergic mechanism. GLT-1, an astrocyte-specific excitatory amino acid transporter, which is responsible for glutamate homeostasis in the brain^37^. It had been previously demonstrated that downregulation of GLT-1 expression in the NAc was associated with continuous exposure to addicting drugs^50–52^. Interestingly, GLT-1 expression was found to be downregulated instantly in cocaine self-administration model^53^. Of note, the glutamatergic transmission is amplified as a result of increase in glutamate concentrations and decrease in glutamate uptake in the synapses^54^. Additionally, it has been observed that glutamate receptors such as mGlu-5 and N-Methyl-D-aspartate could be potentiated and activated by the spillover of glutamate which enhances drug seeking behavior^54^. In the present study, our results suggest that pregabalin at higher doses [60 mg, and 90 mg] may induce addiction partly by downregulating GLT-1 expression and thereby decreasing glutamate uptake at the synaptic cleft.

Treatment with CEF has been reported to prevent drug seeking behavior caused by, in part, decreased GLT-1 expression in methamphetamine, cocaine, ethanol, nicotine, and heroin dependence^26,41–45^ with the drug seeking associated with glutamate spillover secondary to GLT-1 downregulation^25,55–57^. Additionally, the normalization of GLT-1 expression, by CEF treatment, was associated with a decrease in drug-seeking behavior^58,59^. Therefore, pregabalin seeking at the addictive doses of [60 mg, and 90 mg] might be mediated by altering GLT-1 expression as the drug seeking effects of pregabalin was eliminated by CEF pretreatment in the present study. CEF has been demonstrated to have neuroprotective efficacy in many neurological disorders^60,61^ and can offer neuroprotective effects in drug addiction associated with glutamate excitotoxicity^60,62,63^. CEF can freely pass the blood brain barrier and enter the central nervous system to up-regulate GLT-1 making it an attractive potential therapeutic for future clinical use in antagonizing pregabalin-induced drug-seeking behavior^60,62–64^.

One limitation of the present study is that we did not demonstrate a mechanisms of action for CEF in antagonism of pregabalin-induced drug-seeking behavior. GLT-1 plays a central role in inflammatory mechanisms in the brain, which has previously been demonstrated to be associated with drug addition^65–67^. It has been previously reported that central administration of some neurotoxicants causes significant impairement in motor functions, increased neuroinflammation and increased drug addiction^68–70^. Post-treatment with CEF (200 mg/kg) significantly antagonized motor impairment, attenuated lipid peroxidation, restored endogenous antioxidant enzymes glutathione peroxidase and catalase, and decreased drug addiction^71,72^. Taken together, CEF-mediated antagonism of pregabalin-induced drug seeking like effects could promote restoration of glutamate homeostasis, and in this way modulate drug-seeking behavior. CEF could serve as a lead compound for developing treatment for pregabalin abuse as other pharmacological effects of this antibiotic could not be excluded.

Future studies are needed to investigate a mechanistic role for neuroinflamation in pregabalin abuse, as well as sex- and age-related mechanisms in pregabalin-induced neurochemical changes.
